# Combination therapy with dendritic cells and lenalidomide is an effective approach to enhance antitumor immunity in a mouse colon cancer model

**DOI:** 10.18632/oncotarget.15917

**Published:** 2017-03-06

**Authors:** Manh-Cuong Vo, Thanh-Nhan Nguyen-Pham, Hyun-Ju Lee, Thangaraj Jaya Lakshmi, Seoyun Yang, Sung-Hoon Jung, Hyeoung-Joon Kim, Je-Jung Lee

**Affiliations:** ^1^ Research Center for Cancer Immunotherapy, Chonnam National University Hwasun Hospital, Hwasun, Jeollanamdo, Republic of Korea; ^2^ Department of Hematology-Oncology, Chonnam National University Hwasun Hospital, Hwasun, Jeollanamdo, Republic of Korea

**Keywords:** lenalidomide, dendritic cells, mouse colon cancer

## Abstract

In this study, we investigated efficacy of lenalidomide in combination with tumor antigen-loaded dendritic cells (DCs) in murine colon cancer model. MC-38 cell lines were injected subcutaneously to establish colon cancer-bearing mice. After tumor growth, lenalidomide (50 mg/kg/day) was injected intraperitoneally on 3 consecutive days in combination with tumor antigen-loaded DC vaccination on days 8, 12, 16, and 20. The tumor antigen-loaded DCs plus lenalidomide combination treatment exhibited a significant inhibition of tumor growth compared with the other groups. These effects were associated with a reduction in immune suppressor cells, such as myeloid-derived suppressor cells and regulatory T cells, with the induction of immune effector cells, such as natural killer cells, CD4^+^ T cells and CD8^+^ T cells in spleen, and with the activation of cytotoxic T lymphocytes and NK cells. This study suggests that a combination of tumor antigen-loaded DC vaccination and lenalidomide synergistically enhanced antitumor immune response in the murine colon cancer model, by inhibiting the generation of immune suppressive cells and recovery of effector cells, and demonstrated superior polarization of Th1/Th2 balance in favor of Th1 immune response. This combination approach with DCs and lenalidomide may provide a new therapeutic option to improve the treatment of colon cancer.

## INTRODUCTION

Colorectal cancer (CRC) is one of the most common malignancies. Reportedly, CRC is the third most common cancer in males and second in females worldwide, and approximately 10% of all malignancies are colorectal tumors [1]. Although surgery is the primary therapeutic modality for this disease, patients with advanced diseases or cancer recurrence after surgery remain difficult to cure despite the innovative therapeutic options with new chemotherapeutic agents [2–6].

Dendritic cells (DCs) are commonly used to induce therapeutic immunity against cancer. DCs are the most potent antigen-presenting cells (APCs) that are capable of recognizing, processing and presenting tumor cells and thus play a central role in various immunotherapy protocols to generate cytotoxic T lymphocytes (CTLs) [7–15].

In our previous studies, we used DC-specific antigens to treat colon cancer and showed that DC treatment is effective for reducing the tumor mass [16, 17]. Recent studies demonstrated that a DC-based vaccine for colon cancer improved the clinical response [8]. However, based on the results, the clinical efficacy remains inconclusive and should be validated in larger prospective, randomized and controlled studies [18].

Lenalidomide (Revlimid^®^, Celgene Corporation) is an immunomodulatory agent with antiangiogenic and antineoplastic properties, demonstrated efficacy, and an acceptable toxicity profile in multiple myeloma and myelodysplastic syndromes [19]. Lenalidomide was shown to reduce tumor growth significantly and cause necrosis in tumors in a CRC mouse model [20]. In mice, daily administration of 1enalidomide reduced the rate of tumor growth significantly, and histological analysis showed vast areas of necrotic tissue in tumors [20]. Reddy et al. showed that *in vitro* exposure of DCs to lenalidomide resulted in increased levels of IFN-ȶ, TNF-α and MCP-1 compared with the control (DCs only) [21]. Additionally, lenalidomide reduced the proportion and inhibited the function of regulatory T cells (Tregs) [22]. Preclinical data showed that lenalidomide is an active agent against metastatic CRC through modulation of the tumor microenvironment and angiogenesis [23, 24]. Clinical studies have extended the therapeutic effectiveness of lenalidomide against CRC and gastric carcinoma with safety and high efficacy [25].

Therefore, in this study, we investigated whether lenalidomide combined with DC vaccination exerted a synergistic effect in a colon cancer model. This study demonstrated that tumor antigen -loaded DC vaccination with lenalidomide enhanced antitumor immunity in a mouse colon cancer model by inhibiting immune-suppressive cells as well as the recovery of effector cells and demonstrated superior polarization of the Th1/Th2 balance in favor of the Th1 immune response. Our study provides the framework for understanding the role of DCs combined with lenalidomide to inhibit tumor cell growth and restore immune function in colon cancer-bearing mice.

## RESULTS

### Effect of various lenalidomide doses on MC-38 cell viability

**Figure 1:**
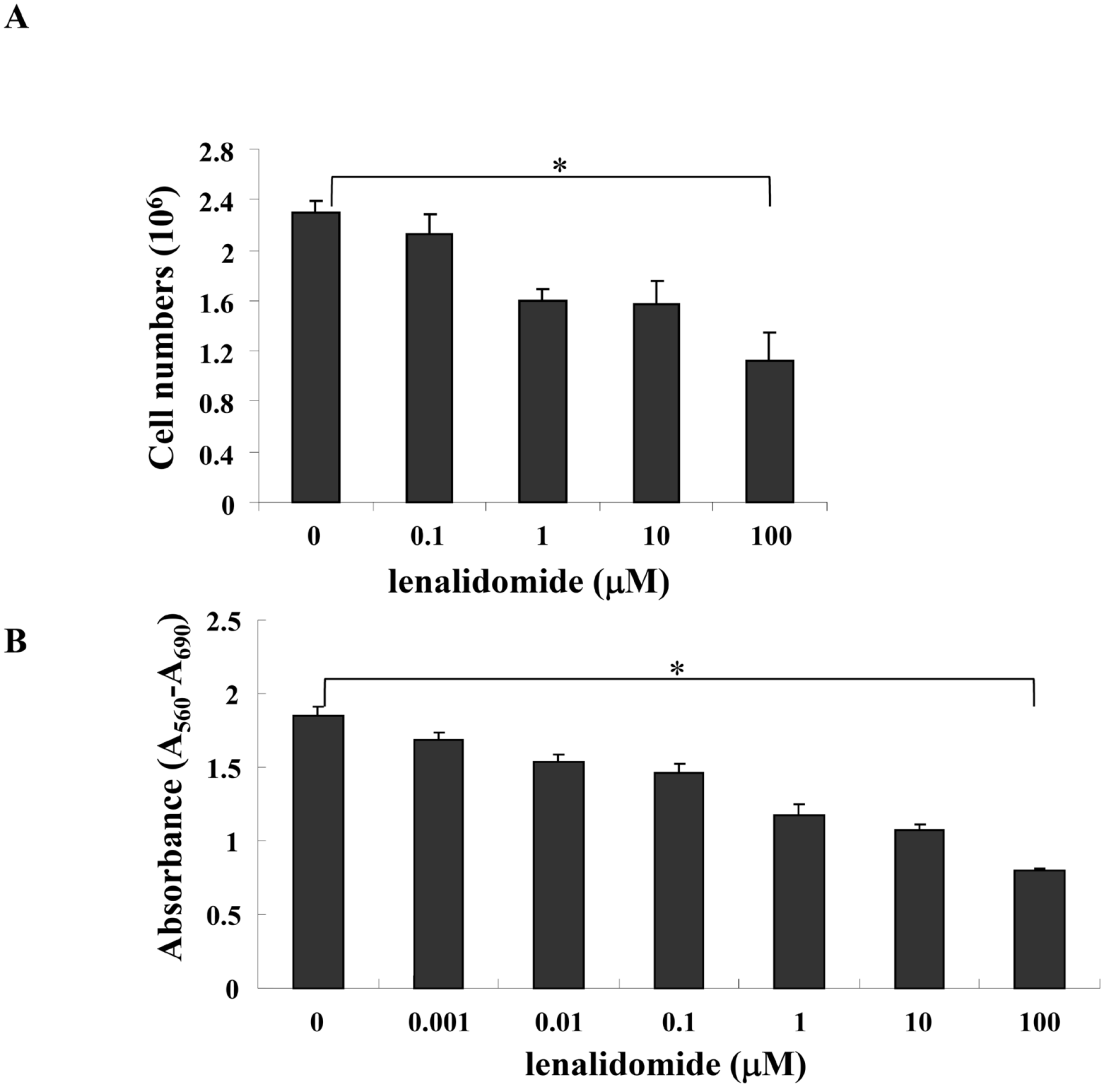
Viability of MC-38 cell lines cultured with various doses of lenalidomide At the start of MC-38 cell line culturing, various doses (0.1, 1, 10 and 100 μM) of lenalidomide were added, and cell viability was evaluated after 2 days. Harvested cells were stained with 0.4% trypan blue **(A)**, and viability was evaluated by 3-(4,5-dimethylthiazol-2-yl)-2,5-diphenyltetrazolium bromide (MTT) assay **(B)**. The results showed significantly decreased survival of MC38 cell lines with an increasing lenalidomide concentration (* *P* < 0.05). The data are representative of three independent experiments.

At the start of MC-38 cell culturing, lenalidomide was added at doses of 0.1, 1, 10 and 100 μM. Cell viability was evaluated after 2 days; harvested cells were stained with 0.4% trypan blue staining (Figure 1A) or evaluated by 3-(4,5-dimethylthiazol-2-yl)-2,5-diphenyltetrazolium bromide (MTT) assay (Figure 1B). The data showed that the survival of MC-38 cell lines was significantly decreased with increasing lenalidomide concentration.

### Synergistic antitumor immunity in a mouse colon cancer model observed after tumor antigen-loaded dendritic cells (DC) vaccination combined with lenalidomide

**Figure 2:**
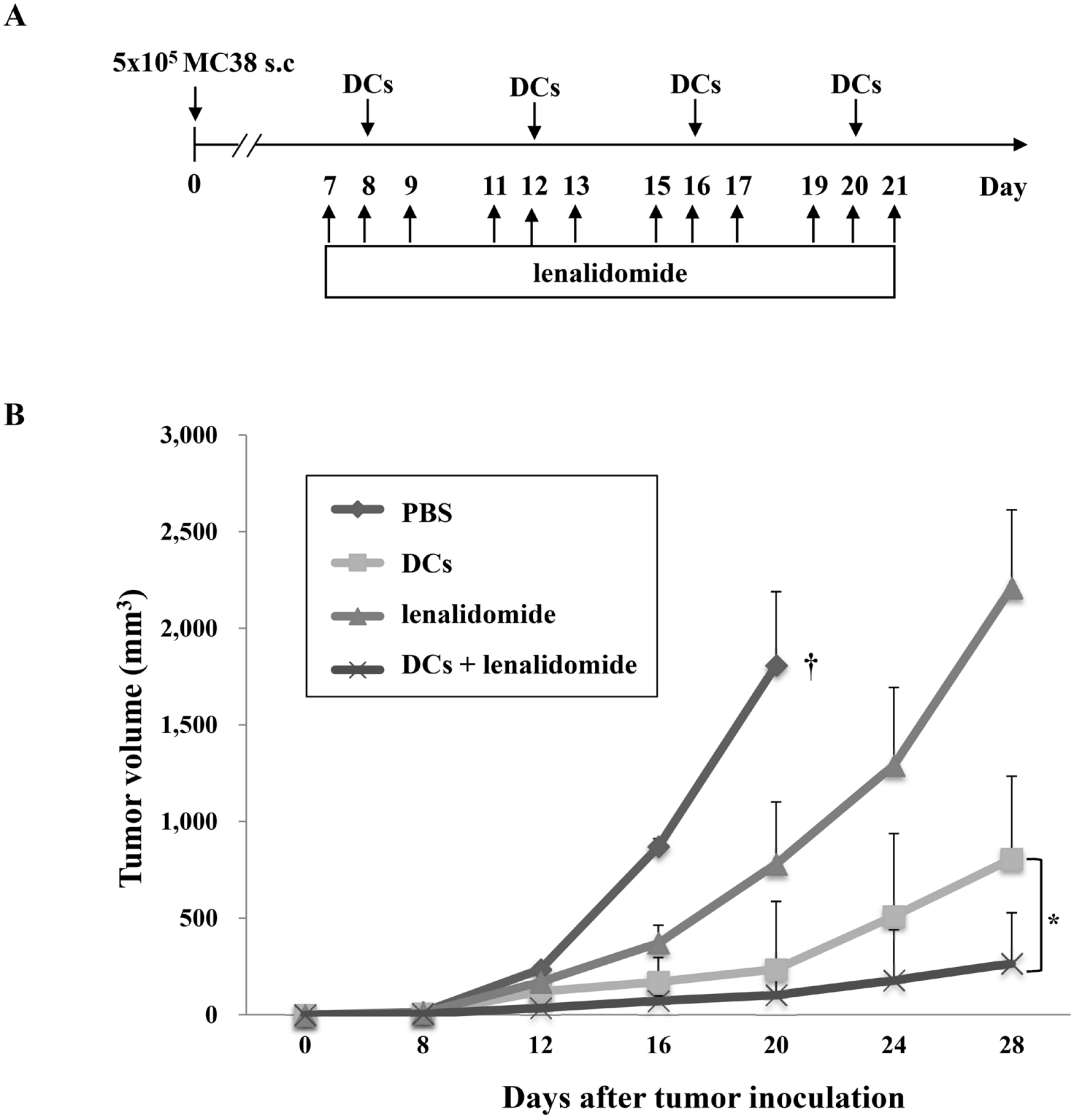
*In vivo* animal vaccination On day 0, MC-38 cells (5 × 10^5^/mouse) were injected subcutaneously into the right flank of C57BL6 mice. **(A)** Schematic representation of the combination of tumor antigen-loaded DCs plus lenalidomide. The following four groups were evaluated: PBS control, lenalidomide injection, tumor antigen-loaded-DC vaccination and tumor antigen-loaded-DC vaccination plus lenalidomide injection. After tumor growth, lenalidomide was injected intraperitoneally on 3 consecutive days to enhance the effect of tumor antigen-loaded DC vaccination. Each dose of tumor antigen-loaded-DCs (1 × 10^6^/mouse) was injected subcutaneously into the left flank of C57BL6 mice in a volume of 0.1 mL PBS at 4-day intervals (on days 8, 12, 16, and 20). **(B)** The data are means ± standard errors of the means and are representative of two independent experiments. The combination of tumor antigen-loaded DCs plus lenalidomide significantly inhibited tumor growth (*, *P* < 0.05 on days 16, 20 and 28) and induced a long-term systemic anti-colon cancer immune response (28 days). Experiments consisted of five mice per group.

To examine whether the immunomodulatory effect of lenalidomide enhanced the efficacy of tumor antigen-loaded DC vaccination, we combined DC vaccination with lenalidomide injection (Figure 2A) to inhibit tumor growth. Tumor-bearing mice vaccinated with PBS or lenalidomide did not show inhibited tumor growth, thus resulting in sacrifice within 3 weeks. In contrast, the tumor-bearing mice vaccinated with tumor antigen-loaded DCs and tumor antigen-loaded DCs plus lenalidomide showed significantly greater inhibition of tumor growth compared with the PBS control or lenalidomide alone. Treatment with the combination of tumor antigen-loaded DCs plus lenalidomide showed significantly greater inhibition of tumor growth compared with tumor antigen-loaded DCs alone (*P* < 0.05; Figure 2B). These results indicated that tumor antigen-loaded DC vaccination in combination with lenalidomide can induce an antitumor immune response against colon cancer.

### Activation of CTLs and natural killer (NK) cells by vaccination with tumor antigen-loaded DCs plus lenalidomide

**Figure 3:**
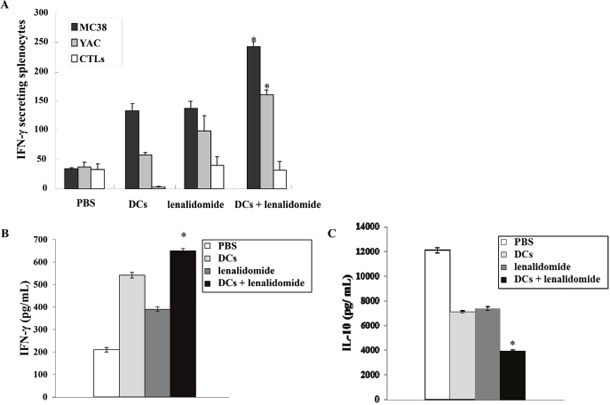
Activation of CTLs and NK cells and cytokine production induced by vaccination with tumor antigen-loaded DCs plus lenalidomide **(A)** The number of IFN-γ secreting lymphocytes in spleens of mice treated with PBS, lenaldiomide, tumor antigen-loaded DCs, and tumor antigen-loaded DCs plus lenalidomide were counted using the IFN-γ ELISPOT assay. DC vaccination combined with lenalidomide injection significantly increased the number of IFN-γ-secreting lymphocytes targeting MC-38 and YAC-1 cells compared with the other groups, indicating the antitumor immune response exhibited both CTL- and NK cell-mediated activities (*, *P* < 0.05). **(B)** IFN-γ and **(C)** IL-10 production from the splenocytes of vaccinated mice was evaluated by ELISA. The culture supernatants of splenocytes from the mice vaccinated with tumor antigen-loaded DCs plus lenalidomide produced lower levels of IL-10 and higher levels of IFN-γ compared with the other mice (* *P* < 0.05). Data shown are means (pg/mL) ± standard deviation (SD) of triplicate cultures from three independent experiments.

**Figure 4:**
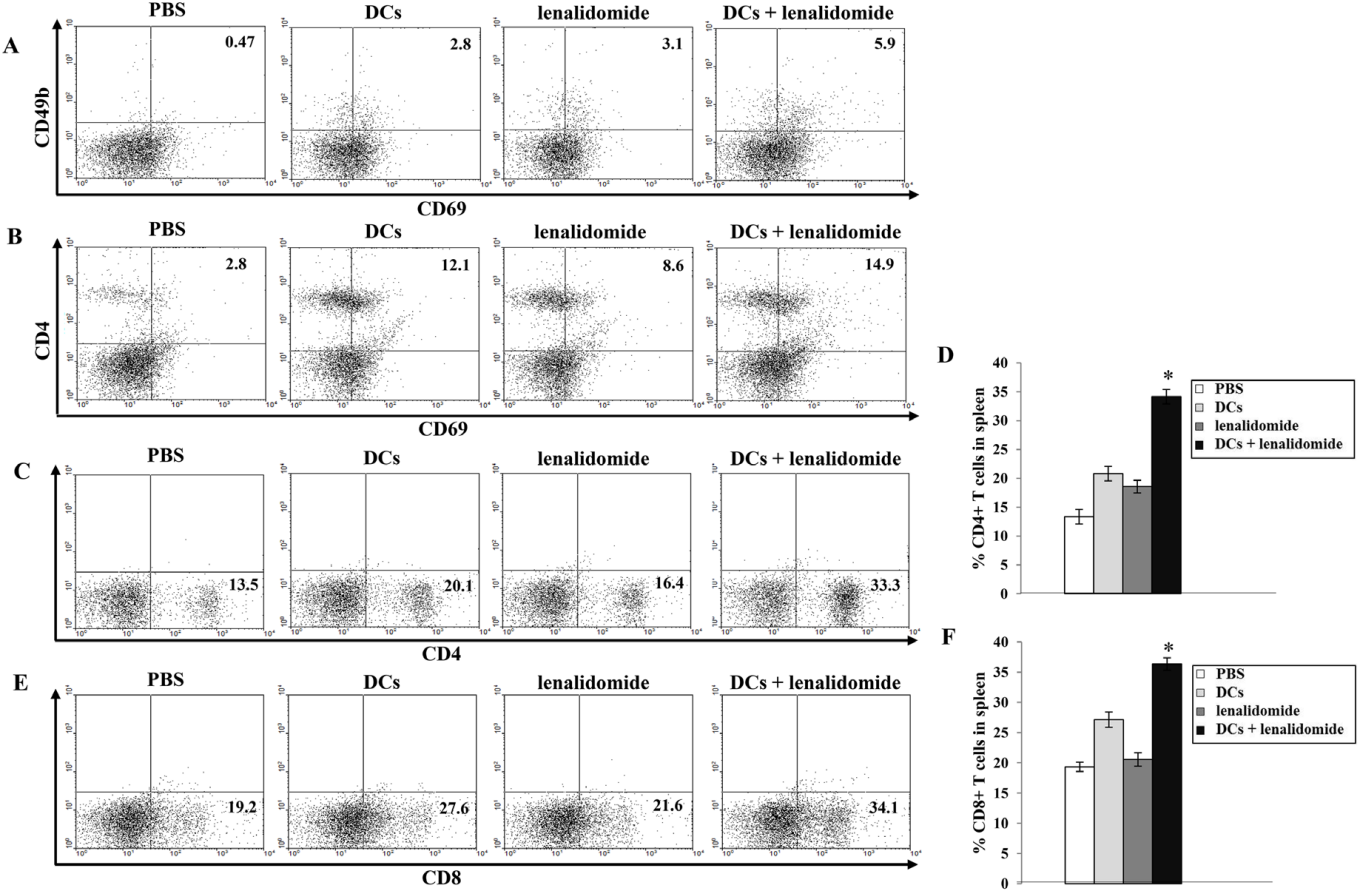
Induction of NK cells, CD4^+^ T cells and CD8^+^ T cells in the spleens of mice treated with a combination of tumor antigen-loaded DCs plus lenalidomide The proportions of NK cells **(A)** measured by flow cytometry. The tumor antigen-loaded DCs plus lenalidomide combination group exhibited the highest proportions of NK cells. Data shown are from one representative experiment. The proportions of CD4^+^ T cells **(B–D)** and CD8^+^ T cells (**E** and **F**) measured by flow cytometry were significantly increased in the tumor antigen-loaded DCs plus lenalidomide combination group compared with the other groups (*, *P* < 0.05). The data are representative of three independent experiments.

To investigate the immunological response of CTLs to tumor antigen-loaded DC vaccination in the mouse MC-38 colon cancer model, splenocytes from each group of vaccinated mice were prepared for IFN-γ ELISPOT assays. MC-38 and YAC-1 cells were used as the target cells. Compared with the PBS control, vaccination with tumor antigen-loaded DCs or tumor antigen-loaded DCs plus lenalidomide led to a significant increase in IFN-γ-secreting splenocytes against MC-38 cells. Interestingly, the antitumor effect of tumor antigen-loaded DCs plus lenalidomide showed the highest number of IFN-γ-secreting splenocytes against MC-38 cells and YAC-1 cells compared with the PBS control, tumor antigen-loaded DCs or lenalidomide alone (*P* < 0.05; Figure 3A). These results indicated that the tumor-inhibitory effects of tumor antigen-loaded DCs plus lenalidomide resulted from the CTL-mediated response as well as the NK cell-mediated responses. In this study, vaccination with tumor antigen-loaded DCs plus lenalidomide led to the highest IFN-γ (Figure 3B) and lowest IL-10 (Figure 3C) production compared with the PBS control, tumor antigen-loaded-DCs or lenalidomide alone. These results suggest that the combination of tumor antigen-loaded DCs plus lenalidomide enhanced Th1 immune responses in addition to tumor-specific CTL responses. Regarding tumor-specific immune responses, tumor antigen-loaded DCs plus lenalidomide showed significantly increased percentages of NK cells (Figure 4A), CD4^+^ T cells (Figures 4B–4D) and CD8^+^ T cells (Figure 4E and 4F) compared with the other groups.

### Efficient inhibition of myeloid-derived suppressor cells (MDSCs) and Tregs due to tumor antigen-loaded DC vaccination plus lenalidomide

**Figure 5:**
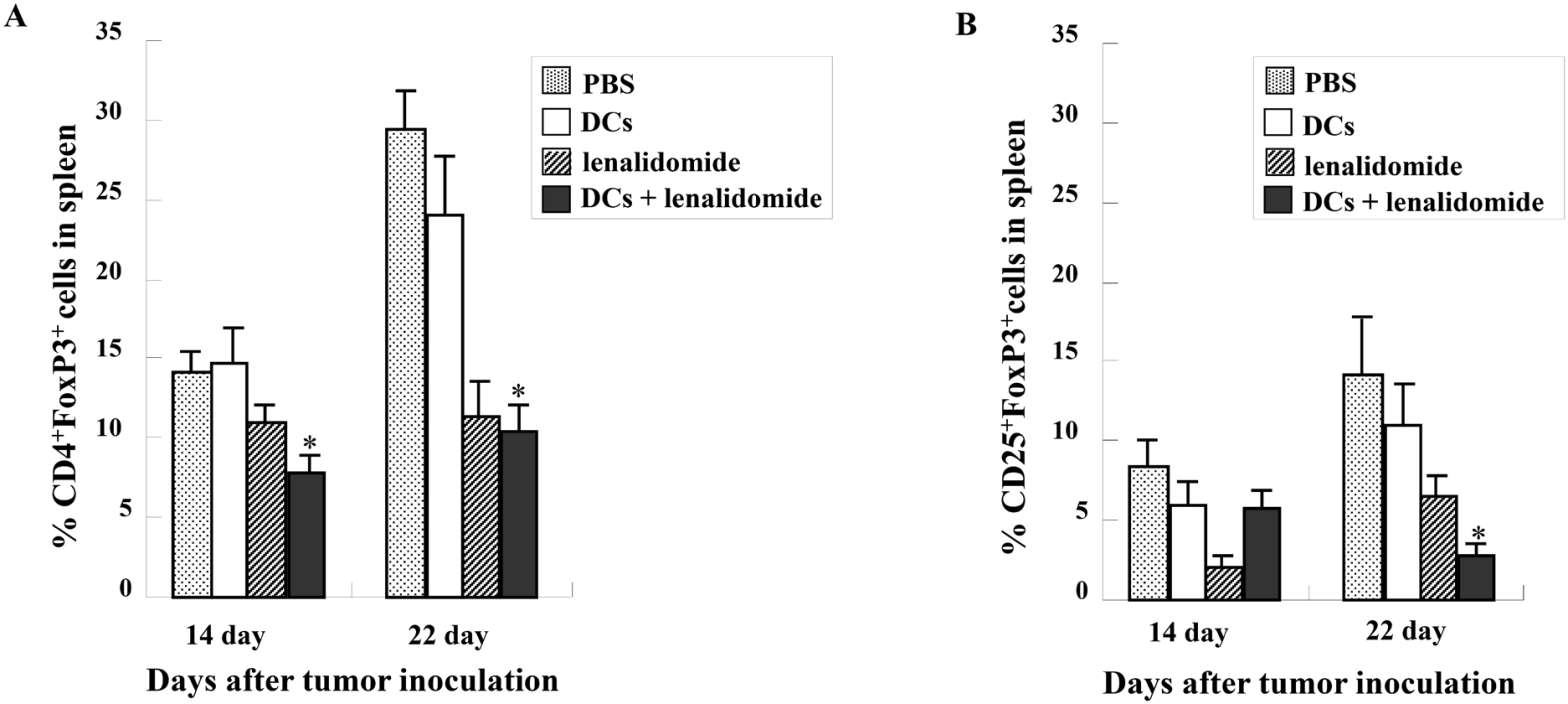
The proportions of Tregs in the spleens of vaccinated tumor-bearing mice measured by flow cytometry The proportions of Tregs (**A** and **B**) were significantly increased in the PBS control and tumor antigen-loaded DC groups compared with the groups injected with lenalidomide after tumor inoculation. The tumor antigen-loaded DC vaccination plus lenalidomide combination group showed significantly decreased proportions of splenic Tregs on days 14 and 22 (*, *P* < 0.05). Data are representative of more than three experiments.

To investigate the immunological mechanisms underlying the enhanced tumor-specific immune response, the effects of combination therapy on the proportions of MDSCs (CD11b^+^Gr1^+^) and Tregs (CD4^+^FoxP3^+^ and CD25^+^FoxP3^+^ cells) were analyzed. The percentages of MDSCs (Supplementary Figure 1) were dramatically reduced in all treatment groups compared with the PBS control group. The tumor antigen-loaded DCs plus lenalidomide combination group exhibited the lowest proportions of splenic MDSCs on days 14, 22 and 32 after tumor inoculation. The proportions of Tregs (Figure 5A and 5B) were significantly increased in the PBS control and tumor antigen-loaded DC groups compared with the groups injected with lenalidomide on days 14 and 22 after tumor inoculation. The tumor antigen-loaded DCs plus lenalidomide combination group exhibited the lowest proportion of splenic Tregs (*P* < 0.05). These findings suggested that tumor antigen-loaded DCs plus lenalidomide enhanced the therapeutic antitumor immunity by inhibiting the immunosuppressive cells in the tumor microenvironment during the vaccination phases.

### Enhancement of phenotypic maturation of splenic DCs due to lenalidomide treatment and inhibition of inhibitory cytokine production due to vaccination with tumor antigen-loaded DCs plus lenalidomide

**Figure 6:**
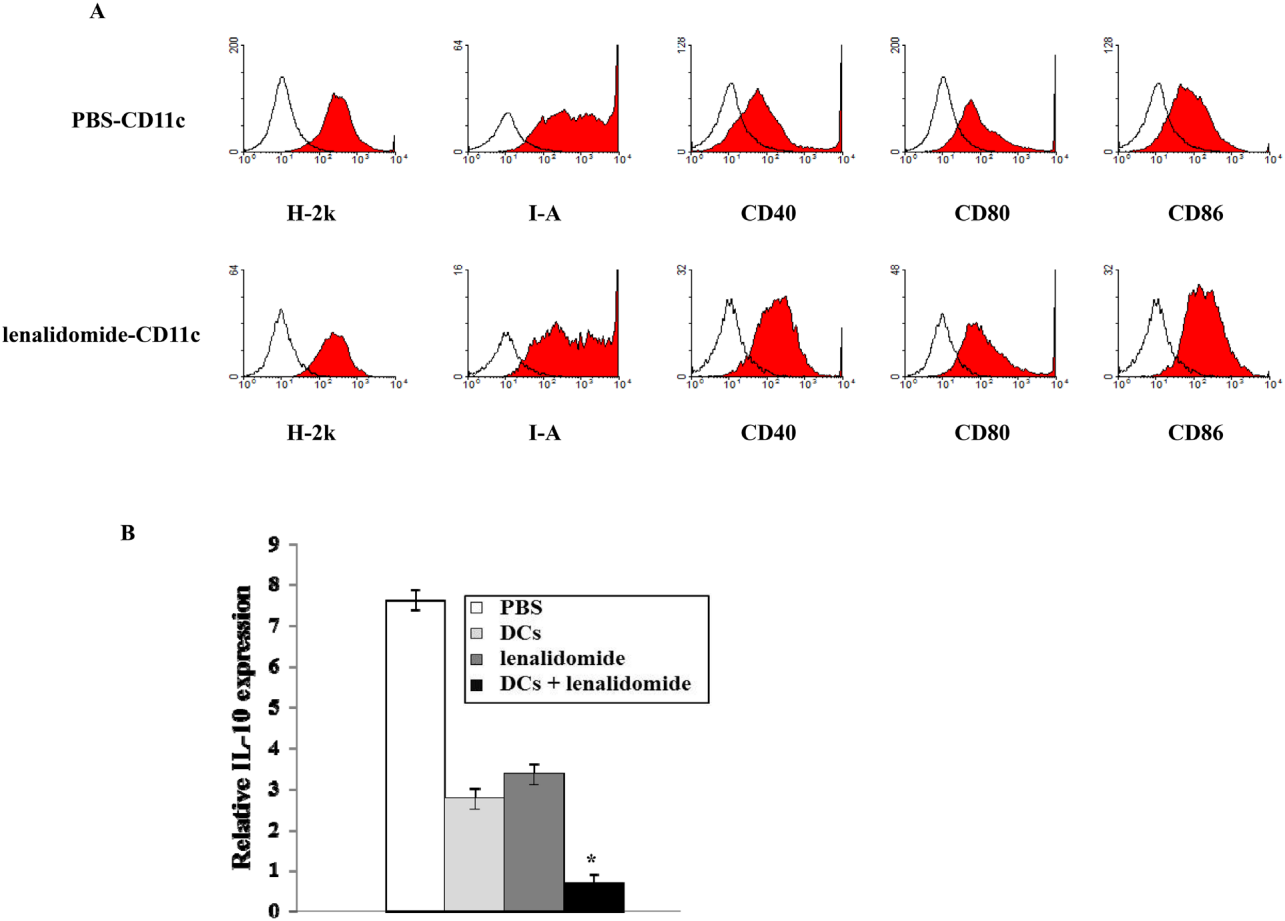
Enhancement of phenotypic maturation of splenic DCs due to lenalidomide treatment and inhibition of inhibitory cytokine production resulting from vaccination with tumor antigen-loaded DCs plus lenalidomide **(A)** The DCs from splenocytes isolated from the mice injected with lenalidomide showed increased expression of H-2k, I-A, CD40, CD80 and CD86 compared with the PBS control group. Representative histograms show the expression of markers (shaded) compared with that in the isotype control (black line). The data shown are from one representative experiment. **(B)** IL-10 gene expression measured by RT- PCR assay was significantly decreased in the tumor antigen-loaded DCs plus lenalidomide combination therapy group compared with the other groups (*, *P* < 0.05). Data are representative of more than three experiments.

To examine the tumor-specific immune response, the effects of lenalidomide on DC maturation were evaluated in injected mice. The DC phenotypes in the spleens of tumor-bearing mice were measured using FACS. The DCs from splenocytes isolated from the mice injected with lenalidomide alone showed increased expression of H-2k, I-A, CD40, CD80 and CD86 compared with the PBS control group (Figure 6A). Regarding the immunological mechanisms underlying the observed enhancement of the tumor-specific immune response, we evaluated the effects of tumor antigen-loaded DCs plus lenalidomide combination therapy on inhibitory cytokine IL-10 production. The IL-10 mRNA levels in the tumors of tumor-bearing mice were measured by RT-PCR (Figure 6B). IL-10 gene expression was significantly decreased in the tumor antigen-loaded DCs plus lenalidomide combination therapy groups compared with the PBS control group, suggesting the tumor antigen-loaded DCs plus lenalidomide combination therapy suppressed the production of inhibitory cytokine IL-10 in mouse tumors.

## DISCUSSION

Lenalidomide is approved by the US Food and Drug Administration for the treatment of myelodysplastic syndromes and multiple myelomas, and it reportedly has activities toward chronic lymphocytic leukemia and non-Hodgkin's lymphoma [26, 27]. A clinical trial by Siena et al. [28] showed the efficacy and safety of a combination treatment with lenalidomide and cetuximab in *KRAS* (v-Ki-ras2 Kirsten rat sarcoma viral oncogene homolog)-mutant metastatic colorectal cancer patients and implicated lenalidomide as an activator of T cells, which may play a role in its antitumor activity. Additionally, the down-regulation of B cell counts by lenalidomide may be linked to its activity in B cell malignancies. In our previous study, lenalidomide synergistically enhanced the anticancer immunity of DC vaccination in a murine multiple myeloma (MM) model by inhibiting immunosuppressor cells, stimulating effector T and NK cells and inducing strong Th1-type immunity [29].

In this study, we demonstrated that lenalidomide can be used as a potential immunomodulatory drug with tumor antigen-loaded DCs in a colon cancer model, augmenting the ability of DC immunization to enhance the magnitude and function of tumor antigen-specific effector CD8^+^ T cells, while also amplifying NK cell function and number. Thus, we considered that the use of lenalidomide may enhance the tumor microenvironment and danger signals for generating potent Th1-type immune responses and antitumor immunity. Recently, several DC-based vaccines using known tumor antigens in mouse colon cancer models have been documented [16, 17, 29–32]. In our study, dying tumor cells were used to load DCs to generate tumor-specific CTLs in tumor-bearing mice. Our data showed that administration of lenalidomide plus tumor antigen-loaded DCs resulted in a dramatic decrease in tumor growth, and we believe that lenalidomide administration will remain efficacious without inducing mortality in this model. IFN-γ ELISPOT assays revealed that lenalidomide plus tumor antigen-loaded DCs resulted in superior induction of tumor-specific CTL-mediated responses against MC-38 tumor cells. Therefore, tumor antigen-loaded DCs plus lenalidomide could be used as an excellent candidate for immunotherapy against colon cancer. The ability of lenalidomide to induce the antitumor efficacy of the DC vaccine may be due to enhancing the proportion of IFN-γ-secreting lymphocytes and suppressing the proportions of Tregs and MDSCs in tumor-bearing mice [29]. Consistently, our results showed that administration of lenalidomide increased the antitumor immune response of the tumor antigen-loaded DC vaccine to eradicate the tumor completely and consequently prolonged the survival of vaccinated mice.

In conclusion, the results of our study suggest that lenalidomide synergistically enhances the effect of DC vaccination by inhibiting the generation of immune suppressive cells as well as the recovery of effector cells during tumor progression. Therefore, the combination therapy with DCs and lenalidomide can be used to improve immunotherapeutic strategy against colon cancer.

## MATERIALS AND METHODS

### Mice and tumor cell lines

Six- to eight-week-old female C57BL6 mice were purchased from Orient Bio (Iksan, Republic of Korea) and maintained under specific pathogen-free conditions. All animal care, experiments and euthanasia were performed in accordance with protocols approved by the Chonnam National University Animal Research Committee. Murine MC-38 colon cancer cell and YAC-1 cell lines were purchased from the American Type Culture Collection (Rockville, MD, USA). The cell lines were maintained in Dulbecco's modified Eagle's medium (DMEM; Gibco-BRL, Grand Island, NY, USA) supplemented with 10% (v/v) fetal bovine serum (FBS) (Gibco-BRL) and 1% (w/v) penicillin/streptomycin (PS).

### Viability of MC-38 cell lines

Cell viability was evaluated after 2 days of culturing. The harvested cells were stained with 0.4% trypan blue (Gibco^TM^, Life Technologies) and examined under a microscope (dead cells were stained and live cells were not stained). In addition, the cell viability was evaluated using the 3-(4,5-dimethylthiazol-2-yl)-2,5-diphenyltetrazolium bromide (MTT) assay (Sigma, St. Louis, MO, USA). MTT solution (20 μL) was added to each well, and the plates were shaken for 10 min. The optical densities of the supernatant were read at 540 nm using a microplate spectrophotometer (Spectra Max 340; Molecular Devices, Sunnyvale, CA, USA).

### Immunomodulatory drugs (IMiDs^®^)

Lenalidomide was donated by Celgene Corporation (Summit, NJ, USA) and dissolved in dimethyl sulfoxide (DMSO) to 100 mg/mL immediately before use. For injection into mice, lenalidomide stock solutions were diluted in sterile 0.9% (v/v) normal saline to a final concentration of 10 mg/mL. The final concentration of DMSO in all experiments was < 0.01% (v/v).

### Generation of bone marrow (BM)-derived DCs

C57BL6 BM-derived immature DCs (imDCs) were generated as described previously [16, 17, 29]. Briefly, BM was harvested from the femurs and tibiae of mice and cultured in RPMI-1640 medium (Gibco-BRL) supplemented with 10% (v/v) FBS (Gibco-BRL) and 1% (w/v) PS in the presence of 20 ng/mL recombinant murine (rm) GM-CSF (R&D Systems, Minneapolis, MN, USA) and 10 ng/mL rmIL-4 (R&D Systems). On culture days 2 and 4, half of the medium was removed and replaced with fresh media containing cytokines. On day 6, imDCs were purified via positive selection with CD11c^+^-magnetic beads (Miltenyi Biotec Inc., Auburn, CA, USA). Then, mature DCs (mDCs) were generated by further cultivation for 48 h of CD11c^+^ DCs with 1 μg/mL lipopolysaccharide (LPS) from *Escherichia coli* (Sigma-Aldrich) and 10 ng/mL rmGM-CSF.

### Generation of tumor antigen-loaded DCs

The generation of tumor antigen-loaded DCs was performed as described previously [29]. Briefly, MC-38 tumor cell death was induced by γ-irradiation (100 Gy) (Gammacell-1000 Elite, MDS Nordion, Canada) followed by overnight culture in RPMI-1640, and the cells were mixed with imDCs 2 h after maturation in a 2:1 ratio (DCs:dead tumor cells).

### Animal vaccination

The following four vaccination groups were established: 1) PBS control, 2) tumor antigen-loaded DC vaccination, 3) lenalidomide injection, and 4) tumor antigen-loaded DC vaccination plus lenalidomide injection. On day 0, mice were injected subcutaneously with 5 × 10^5^ MC-38 cells in a volume of 0.1 mL into the right flank. After tumor growth, lenalidomide (50 mg/kg/day) was injected intraperitoneally over 3 consecutive days to enhance the effect of tumor antigen-loaded DC vaccination. Each tumor antigen-loaded DC vaccine (1 × 10^6^ cells/mouse) was injected subcutaneously into the left flank of C57BL6 mice in a 0.1 mL volume on days 8, 12, 16 and 20. To assess the antitumor status of vaccinated mice, the length, width, and height of each tumor were measured every 3-4 days using a Vernier caliper, and the tumor volume was calculated using the standard formula for the volume of an ellipsoid: V = 4/3π(length × width × height/8).

### Phenotypic analysis of splenocytes from vaccinated mice

At the indicated time points, the mice were sacrificed, and the splenocyte phenotypes were characterized by their cell surface markers using fluorescently labeled monoclonal antibodies (mAbs) and analyzed using flow cytometry. The cells were stained with the following mAbs (eBioscience, San Diego, CA, USA): CD11b-FITC, Gr-1-PE, CD4-PE, CD8-FITC, CD49b-PE, CD44-PE, CD62L-FITC, CD69-FITC and NK1.1-PE. Isotype-matched controls were run in parallel. Cell debris was eliminated by forward- and side-scatter gating. The samples were acquired on a FACS Calibur cell sorter (Becton Dickinson, Mountain View, CA, USA) and the data analyzed using WinMDI ver. 2.9 software (Biology Software Net: http://en.bio-soft.net/other/WinMDI.html).

### Tumor antigen-specific CTLs activities of vaccinated mice

The tumor antigen-specific CTL activities were investigated as described previously [17, 29, 33]. Briefly, splenocytes (1 × 10^6^) isolated from vaccinated mice 7 days after the final immunization (day 27) were added to 24-well plates and restimulated with irradiated MC-38 cells (5 × 10^5^ cells) for 5 days in RPMI-1640 (Gibco-BRL) containing 10% FBS (Gibco-BRL) and 1% PS supplemented with 20 ng/mL rmIL-2 (R&D systems). After restimulation, the splenocytes were assessed for tumor antigen-specific CTLs using a mouse IFN-γ Enzyme-Linked Immunospot (ELISPOT) assay (BD Bioscience). The MC-38 cell line and NK-sensitive YAC-1 cell line were used as target cells.

### *In vitro* analysis of cytokine production from vaccinated mice

Cytokine (IFN-γ and IL-10) production from vaccinated mice was determined using the BD OptEIA^TM^ enzyme-linked immunosorbent (ELISA) assay (BD Bioscience). Supernatant from restimulated splenocytes of vaccinated mice was used to measure the production of Th1- and Th2-polarizing cytokines. Each sample was analyzed in triplicate, and the mean absorbance for each set of standards and samples was calculated.

### Intracellular staining assay of Tregs generated in the spleens of vaccinated mice

To evaluate the proportion of Tregs, 1 × 10^6^ splenocytes from vaccinated mice were harvested and stained with PE-conjugated CD4 and FITC-conjugated CD25 antibodies for 30 min at 4°C. Fc block was added before incubation with surface antibodies. Then, the cells were washed and permeabilized with FACSTM Permeabilizing Solution 2 (BD Bioscience) for 30 min at room temperature. After washing twice, the cells were stained with an Alexa Fluor-conjugated Foxp3 antibody (Miltenyi Biotec) for 30 min at 4°C. The samples were acquired on a FACS Calibur cell sorter (Becton Dickinson) and the data analyzed using WinMDI Ver. 2.9 software.

### Measurement of IL-10 mRNA production using RT-PCR

Total RNA was extracted from the tumors of tumor-bearing mice (day 27) using TRIzol reagent (Invitrogen, Waltham, MA, USA). RT-PCR was performed using SuperScript III reverse transcriptase (Invitrogen) and oligo-dT primer (Invitrogen) according to the manufacturer's instructions. In brief, 400 ng total RNAs and 1 μL 50 μM oligo-dT were used to synthesize the first strand of cDNA in a total volume of 20 μL. The primer sequences used were as follows: IL-10 reverse primer, 5'-ACCTGCTCCACTGCCTTGCT-3'; IL-10 forward primer, 5'-TGAGGCGCTGTCGTCATCGATTTCTCCC-3'; β-actin transcript levels were used to normalize the amount of cDNA in each sample.

### Statistical analyses

All statistical analyses were performed using SPSS version 13.0 for Windows (SPSS Inc., Chicago, IL, USA). The Mann-Whitney *U* test was performed to detect differences in the non-parametric variables between the groups. A *P*-value < 0.05 was considered to indicate statistical significance.

## SUPPLEMENTARY MATERIALS FIGURES


