# Supplementary material for: Combination therapy with dendritic cells and lenalidomide is an effective approach to enhance antitumor immunity in a mouse colon cancer model
Source: Oncotarget. 2017 Mar 6;8(16):27252–62. doi: 10.18632/oncotarget.15917 (PMC5432332; doi:10.18632/oncotarget.15917)
Supplement: Supplementary file 1 [file oncotarget-08-27252-s001.pdf]

Combination therapy with dendritic cells and lenalidomide is an effective approach to enhance antitumor immunity in a mouse colon cancer model

SUPPLEMENTARY MATERIALS

SUPPLEMENTARY FIGURE

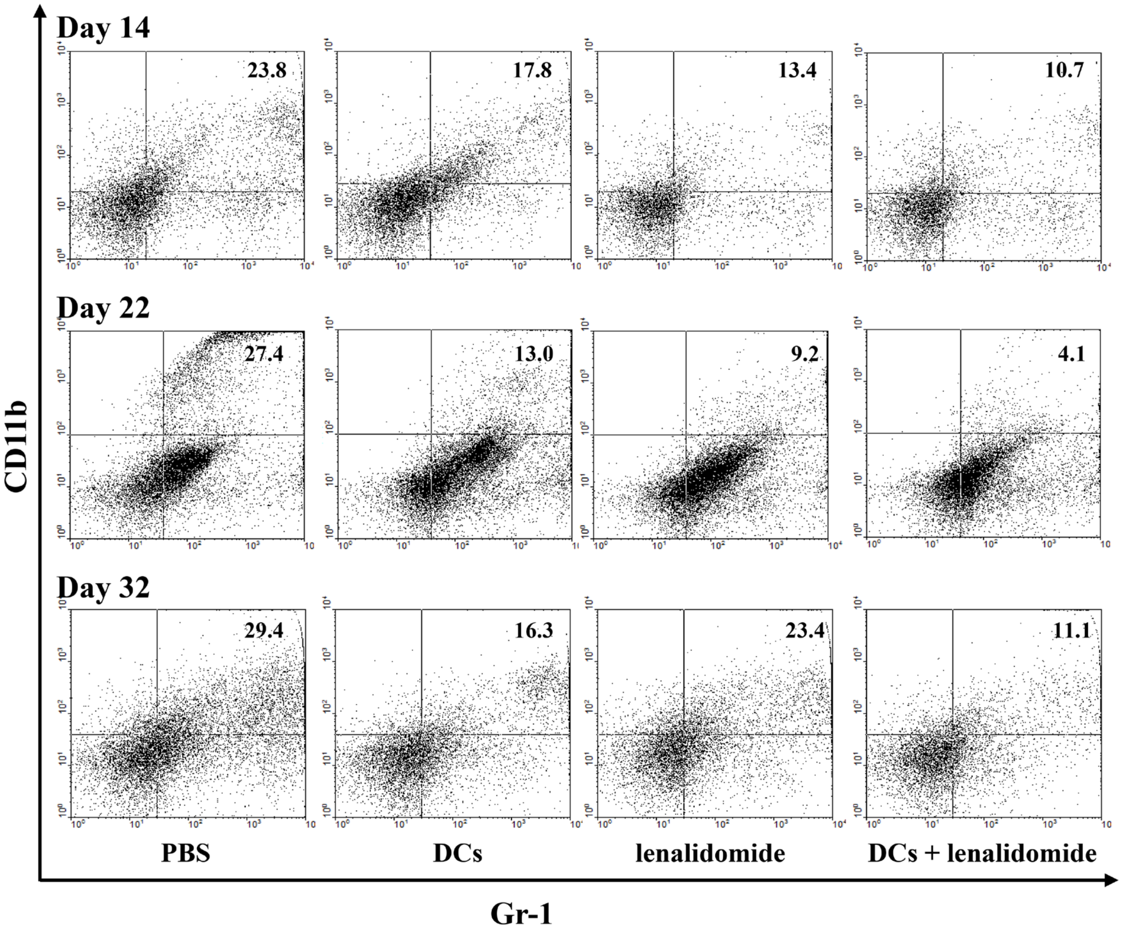

Supplementary Figure 1: The proportions of MDSCs in the spleens of vaccinated tumor-bearing mice measured by flow cytometry. The percentages of MDSCs were increased in the PBS control groups after tumor inoculation. The tumor antigen-loaded DCs plus lenalidomide combination group exhibited the lowest proportions of splenic MDSCs on days 14, 22 and 32. The data shown are from one representative experiment.
